# Climate influence on plant–pollinator interactions in the keystone species *Vaccinium myrtillus*

**DOI:** 10.1002/ece3.8910

**Published:** 2022-05-23

**Authors:** Siri L. Olsen, Marianne Evju, Jens Åström, Jørn O. Løkken, Sondre Dahle, Jonas L. Andresen, Nina E. Eide

**Affiliations:** ^1^ 8019 Norwegian Institute for Nature Research Oslo Norway; ^2^ Faculty of Environmental Sciences and Natural Resource Management Norwegian University of Life Sciences Ås Norway; ^3^ 8019 Norwegian Institute for Nature Research Trondheim Norway; ^4^ University of South‐Eastern Norway Bø Norway

**Keywords:** bilberry, biotic interactions, climate change, elevational gradient, fruit production, seed production

## Abstract

Climate change is altering the world's ecosystems through direct effects of climate warming and precipitation changes but also indirectly through changes in biotic interactions. For instance, climate‐driven changes in plant and/or insect communities may alter plant–pollinator interactions, thereby influencing plant reproductive success and ultimately population dynamics of insect‐pollinated plants. To better understand how the importance of insect pollination for plant fruit set varies with climate, we experimentally excluded pollinators from the partly selfing keystone species *Vaccinium myrtillus* along elevational gradients in the forest‐tundra ecotone in central Norway. The study comprised three mountain areas, seven elevational gradients spanning from the climatically relatively benign birch forest to the colder alpine areas above the tree line, and 180 plots of 1 × 1 m, with experimental treatments allocated randomly to plots within sites. Within the experimental plots, we counted the number of flowers of *V*. *myrtillus* and counted and weighed all fruits, as well as seeds for a selection of fruits. Excluding pollinators resulted in lower fruit production, as well as reduced fruit and seed mass of *V*. *myrtillus*. In the alpine sites pollinator exclusion resulted in 84% fewer fruits, 50% lower fruit weight, and 50% lower seed weight compared to control conditions. Contrary to our expectations, the negative effect of pollinator exclusion was less pronounced in the forest compared to alpine sites, suggesting that the importance of insect pollination for seed production is lower at low elevations. Our findings indicate that the keystone species *V*. *myrtillus* is relatively robust to changes in the pollinator community in a warmer climate, thereby making it less vulnerable to climate‐driven changes in plant–pollinator interactions.

## Graphical abstract

Climate‐driven changes in plant and/or insect communities may alter plant–pollinator interactions in a warming world, thereby influencing plant reproductive success and ultimately population dynamics of insect‐pollinated plants. Our experimental study shows that the plant species *Vaccinium myrtillus* is more robust to changes in the pollinator community in warmer compared to colder climates. This suggests that this keystone species is less vulnerable to climate‐driven changes in plant–pollinator interactions than expected.

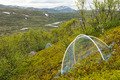

## 1 INTRODUCTION

Climate change is one of the most prominent drivers of global environmental change, impacting both biodiversity and ecosystem functions and services worldwide (IPBES, 2019). Climate change may influence plants and animals through direct responses to temperature and precipitation change, but also indirectly through shifts in biotic interactions (e.g., Adler et al., 2012). Although climate change has been predicted to affect all major types of biotic interactions (Tylianakis et al., 2008), we lack knowledge about the ecological impacts of such changes (e.g., Adler et al., 2012; Brooker, 2006; Gilman et al., 2010).

The interaction between plants and pollinators is fundamental to most terrestrial ecosystems: pollinators facilitate plant reproduction, while plants provide food resources for the pollinators. Pollen limitation, i.e. reduced plant reproduction due to limited pollen availability, is widespread in natural ecosystems (e.g., Bennett et al., 2020; Burd, 1994; Knight et al., 2005). Accordingly, Rodger et al. (2021) conclude that an absence of pollinators would strongly reduce reproduction by seed for 50% of flowering plants, and that one‐third of these species are completely dependent on pollinators for seed production. This suggests that animal‐pollinated plants are vulnerable to changes in plant–pollinator interactions.

The plant–pollinator interaction has been predicted to be sensitive to climate change, especially through spatial and temporal mismatches between plants and their pollinators (e.g., Memmott et al., 2007). Although many studies conclude that plant–pollinator interactions are relatively robust to climate‐driven alterations (Forrest, 2015; Hegland et al., 2009; Rafferty, 2017), such mismatches have been documented (e.g., Burkle et al., 2013; Pyke et al., 2016; Robbirt et al., 2014) and are likely to become more frequent with the forecasted climate warming, potentially resulting in reduced plant reproductive success.

Alpine ecosystems are considered to be especially sensitive to climate warming (e.g., IPCC, 2014; Theurillat & Guisan, 2001), and upward shifts in elevation of plant species are already observed on mountain summits throughout Europe (e.g., Grabherr et al., 1994; Pauli et al., 2012; Steinbauer et al., 2018), accompanied by a shift in species composition (Gottfried et al., 2012). Insect communities show similar patterns of upward shifts coupled with changes in species composition (Fourcade et al., 2019; Franzén & Öckinger, 2012; Ploquin et al., 2013). However, the effect of these changes in plant and insect communities on the outcome of plant–pollinator interactions, such as plant seed production, remains understudied (Hegland et al., 2009).

The contribution of pollinators to plant seed production has been predicted to be higher in warmer, low‐elevation climates compared to colder, high‐elevation climates due to the scarcity of pollinators (e.g., Arroyo et al., 1985; Lázaro et al., 2015; Totland, 1993) and correspondingly lower levels of cross‐pollination (Billings, 1974; Crawford, 1989) at high elevation. Contrastingly, similar levels of cross‐pollination have been found in low‐elevation and alpine plant populations (Bingham & Orthner, 1998, see also García‐Camacho & Totland, 2009). Moeller et al. (2017) recently demonstrated a global latitudinal gradient in outcrossing but did not examine patterns along elevational gradients. Hence, it remains unclear whether climate‐driven changes in plant–pollinator interactions will have a greater impact on plant seed production in warmer, low‐elevation sites compared to colder, high‐elevation sites.

We used space‐for‐time substitution to examine the importance of pollinators for seed production in a boreal‐alpine plant and explored how this varied with the local climate. We experimentally excluded pollinators from the partly selfing keystone species *Vaccinium myrtillus* along elevational gradients in three mountain areas in central Norway, allowing us to examine how the importance of pollinators for seed production varies with temperature. Such manipulation of pollinator availability along environmental gradients is recommended by Hegland et al. (2009) to assess the effect of climate on plant–pollinator interactions. Specifically, we ask (1) Does the experimental exclusion of pollinators affect fruit production, fruit weight, seed number, and seed mass in *V*. *myrtillus*? and (2) How do the effects of pollinator presence vary with climate?

## 2 MATERIALS AND METHODS

### 2.1 Study area and study species

**FIGURE 1:**
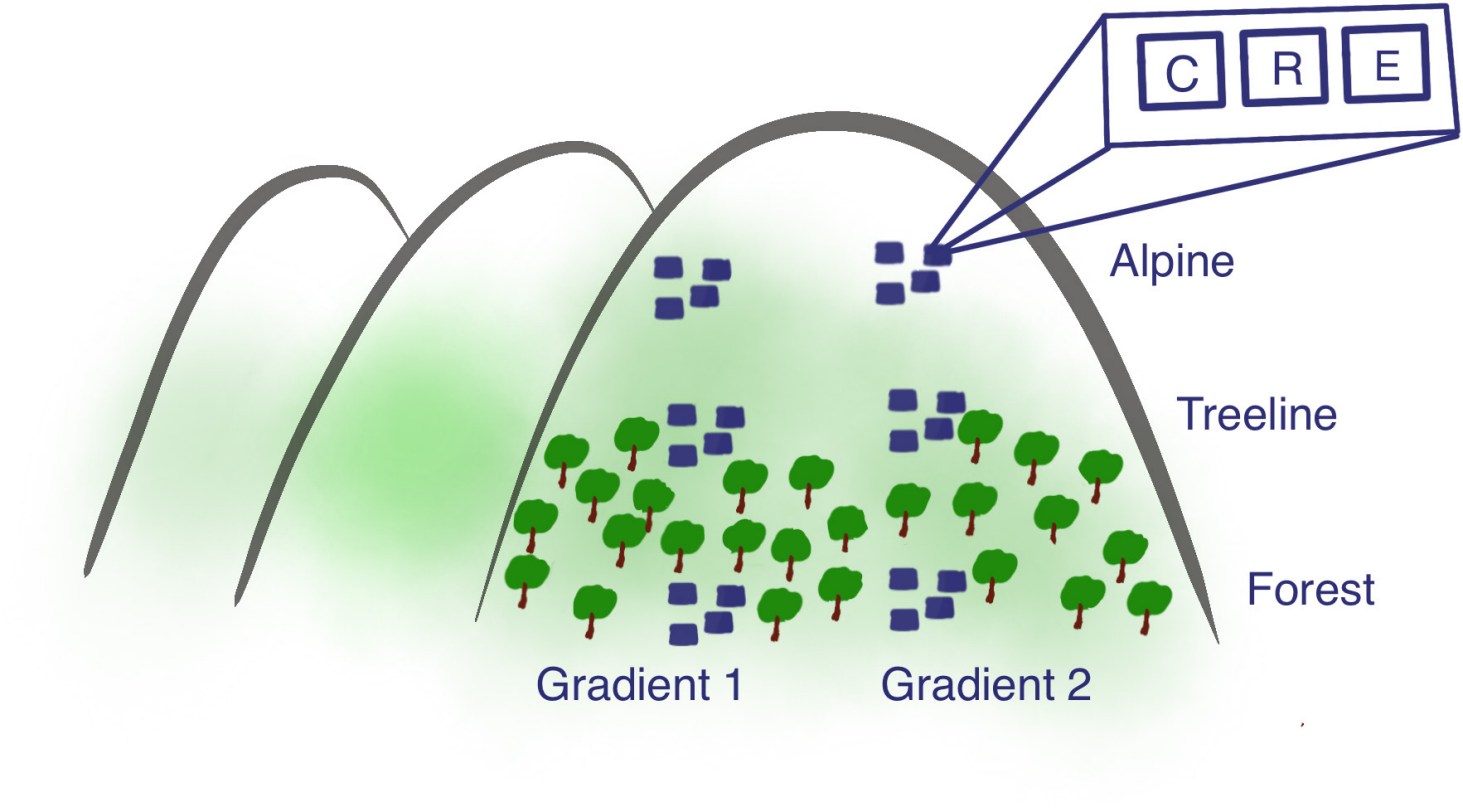
Overview of the experimental design. The study encompassed three mountain areas, each with two elevational gradients consisting of three sites: one in the mountain birch forest, one at the treeline, and one in the alpine tundra. Each site had four experimental blocks, each consisting of three plots, one for each treatment: control (C), pollinator reduction (R), and pollinator exclusion (E)

The study was carried out in 2017 and 2018 in three mountain areas, Forollhogna, Dovrefjell, and Grødalen in Sunndalsfjella, situated along an east‐west gradient in central Norway. Climatic characteristics of the three mountain areas are presented in Nystuen et al. (2014). In each area except Dovrefjell, we established two replicate elevational gradients from the mountain forest to the alpine tundra, and along each gradient we established three experimental sites: one in the mountain birch forest, one at the treeline, and one in the open alpine tundra (Figure 1). The sites were established in heathland vegetation with a high abundance of *V*. *myrtillus*. In Dovrefjell, we only had one gradient due to few *V*. *myrtillus*‐dominated sites. The mean distance between elevational levels was approximately 160 m, and the difference in mean summer temperature was on average 1.0°C between the forest and treeline sites and 0.6 °C between the treeline and alpine sites (Table 1).

**TABLE 1:** Study area, gradient number, site, geographical location (latitude and longitude), elevation (m a.s.l.), mean summer temperature (June–August, °C) (met.no, normal period 1999–2020) and mean cover (%) of *Vaccinium myrtillus* in 2017 and 2018 in 12 1 × 1 m plots for each of the 15 study sites in central Norway

Study area	Gradient	Site	Lat	Long	m a.s.l.	Temp.	% Cover
Forollhogna	1	Forest	62.73981	11.13213	732	9.7	39
Forollhogna	1	Treeline	62.73630	11.11290	838	9.2	22
Forollhogna	1	Alpine	62.73018	11.10128	977	8.4	22
Forollhogna	2	Forest	62.76015	10.93677	834	9.2	26
Forollhogna	2	Treeline	62.77159	10.93898	922	9.0	57
Forollhogna	2	Alpine	62.77283	10.94550	995	8.6	40
Dovrefjell	1	Forest	62.49849	9.64686	821	9.3	51
Dovrefjell	1	Treeline	62.23435	9.50226	1079	8.2	51
Dovrefjell	1	Alpine	62.21469	9.50463	1231	7.4	27
Grødalen	1	Forest	62.53340	8.95921	826	9.4	47
Grødalen	1	Treeline	62.52699	8.93736	1088	8.0	26
Grødalen	1	Alpine	62.52243	8.92815	1201	7.3	21
Grødalen	2	Forest	62.55506	8.94866	746	9.8	53
Grødalen	2	Treeline	62.57567	8.93331	827	9.2	67
Grødalen	2	Alpine	62.58598	8.89399	1037	7.9	29

*V*. *myrtillus* is a keystone species in boreal and low‐alpine areas as it is highly abundant and both the fleshy fruits and vegetative parts provide an important food source for animals (Hegland et al., 2010 and references therein, Selås et al., 2021). This early‐flowering, deciduous, clonal dwarf‐shrub is partly selfing, and its main pollinators are bumblebees, bees, and wasps (Ritchie, 1956), of which bumblebees are dominant in alpine habitats. Andresen (2019) shows that bumblebees do indeed frequently visit and transport pollen from *V*. *myrtillus* in the Grødalen study area and that *V*. *myrtillus* dominates bumblebee pollen loads in the spring.

### 2.2 Experimental design

At each site, we established four blocks, approximately 30–100 m apart (depending on bilberry abundance), each with three 1 × 1 m plots with 3‒5 m distance, resulting in a total of 180 plots (Figure 1). Within each block, we applied three experimental treatments to examine the importance of pollinators for *V*. *myrtillus*: control, pollinator reduction, and pollinator exclusion. Treatments were randomly assigned to the three plots in each block. The pollinator reduction and exclusion treatments were achieved by placing dome‐shaped cages made of two approximately 2.5 m long PVC tubes bent diagonally over the plots (Figure S1), as described by Lundgren et al. (2013). The size of the resulting cages (w × l × h) was approximately 1.5 × 1.5 × 1 m. For the reduction treatment, the cages were covered with berry netting with a mesh size of 1.5 × 1.5 cm, through which at least some pollinators, including bumblebees, were able to enter and exit the plots (Siri L. Olsen & Jørn Olav Løkken, per.obs., 2017), whereas the exclusion cages were covered with insect netting with a mesh size of 2 × 2 mm, which no flying insects were observed to penetrate. The mesh was fastened to the PVC tubes using plastic strips. To prevent non‐flying pollinators from accessing the plots, the netting was fixed to the ground with n‐shaped plugs. Initial analyses after 1 year of treatment suggested that the reduction treatment had a very limited effect, most likely because the mesh size was too large and therefore did not represent a barrier to pollinators. This treatment was therefore discontinued and is not presented here.

At peak flowering time, we counted the number of flowers in each plot. The timing varied along the elevational gradients, with flowering time peaking a week or two earlier in the mountain forest compared to the treeline and alpine sites. When the majority of the fruits were ripe, all fruits from each plot were collected, counted, dried at 60° for 48 h, and weighed. Among the fruits collected in 2017, we randomly picked one mature fruit per plot, and re‐wetted and dissected them before counting the number of seeds under a stereomicroscope. The seeds were subsequently dried at 60° for 48 h and weighed.

The experimental treatments could potentially influence e.g. micro‐climatic conditions within the mesh cages and thereby bias our results. Lundgren et al. (2013) found no biotic or abiotic side‐effects of reduction cages except for a tendency for reduced wind speed. To check for side‐effects of the exclusion cages, we measured temperature using B‐series WatchDog B101 8K temperature loggers (Spectrum Technologies Inc.) and illuminance (lux) using a Hagner EC1 digital luxmeter (B. Hagner AB) in all control and exclusion plots in 2018. All temperature loggers were placed in white plastic boxes to prevent moisture damage, and the boxes were placed at ground level, shaded by the vegetation. The temperature was recorded every fourth hour from mid‐June to mid‐August, whereas illuminance was measured once in each plot at peak flowering, making sure to measure all plots in one site on the same day. Initial analyses showed that both temperature (Figure S2) and illuminance (Figure S3) were lower in the exclusion plots compared to the control plots. However, although Eckerter et al. (2019) show that light availability can influence reproduction in *V*. *myrtillus*, this did not seem to affect *V*. *myrtillus* fruit production. Hand‐pollinating flowers in six separate exclusion plots in one site in 2018 resulted in 18.0 ± 10.9 fruits per plot, while the four “regular” exclusion plots in the same site had 0.0 ± 0.0 fruits in the same year, suggesting that the side‐effects of the exclusion cages did not prevent fruit set (Jonas L. Andresen, unpublished data, 2018).

### 2.3 Statistical analyses

We used a zero‐inflated generalized mixed‐effects model with a negative binomial distribution to test whether the exclusion of pollinators influenced the number of fruits produced by *V*. *myrtillus* and whether the exclusion effect varied with elevation. Treatment (levels: control and exclusion) and elevation level (levels: alpine, treeline, and forest) were used as fixed factors, and we included random intercepts for year and block. Area and gradient had a negligible effect and were therefore not included in the random effects. The zero‐inflation structure included a constant random intercept. Due to initial differences in the number of flowers between treatments, we included the number of flowers (log‐transformed) as an offset in the model, thus analyzing the number of developed fruits per flower. Only plots with flowers were included in the analysis, thereby excluding 15 plots in 2017 and 21 plots in 2018.

Further, we used a generalized mixed‐effects model with a Gaussian distribution to test whether the exclusion of pollinators influenced the mean weight (mg) of *V*. *myrtillus* fruits, and whether this varied with elevation. The fixed and random effects were the same as for the fruit production model. No offset was included. The mean fruit weight was square‐root transformed for the errors to be normally distributed, and only plots with fruits were included in the analysis, thereby excluding 47 plots in 2017 and 80 in 2018.

Finally, we used generalized mixed‐effects models with a negative binomial and Gaussian distribution, respectively, to test whether the exclusion of pollinators influenced the number of seeds per fruit or mean seed weight (mg) in 2017. The fixed effects were the same as for the two previous models, but the only block was used as a random effect. The mean seed weight was log‐transformed for the errors to be normally distributed. Only plots with fruits were included in the analyses, thereby excluding 47 plots, and two exclusion plots with fruits with zero seeds were excluded due to a strong influence on model estimates.

All analyses were performed in R (R Core Team, 2020) using the glmmTMB (Brooks et al., 2017) and DHARMa (Hartig, 2020) packages for the modeling and model diagnostics, respectively. Effect size plots were made using the forestplot package (Gordon & Lumley, 2020), and confidence intervals were obtained using the confint function.

## 3 RESULTS

**FIGURE 2:**
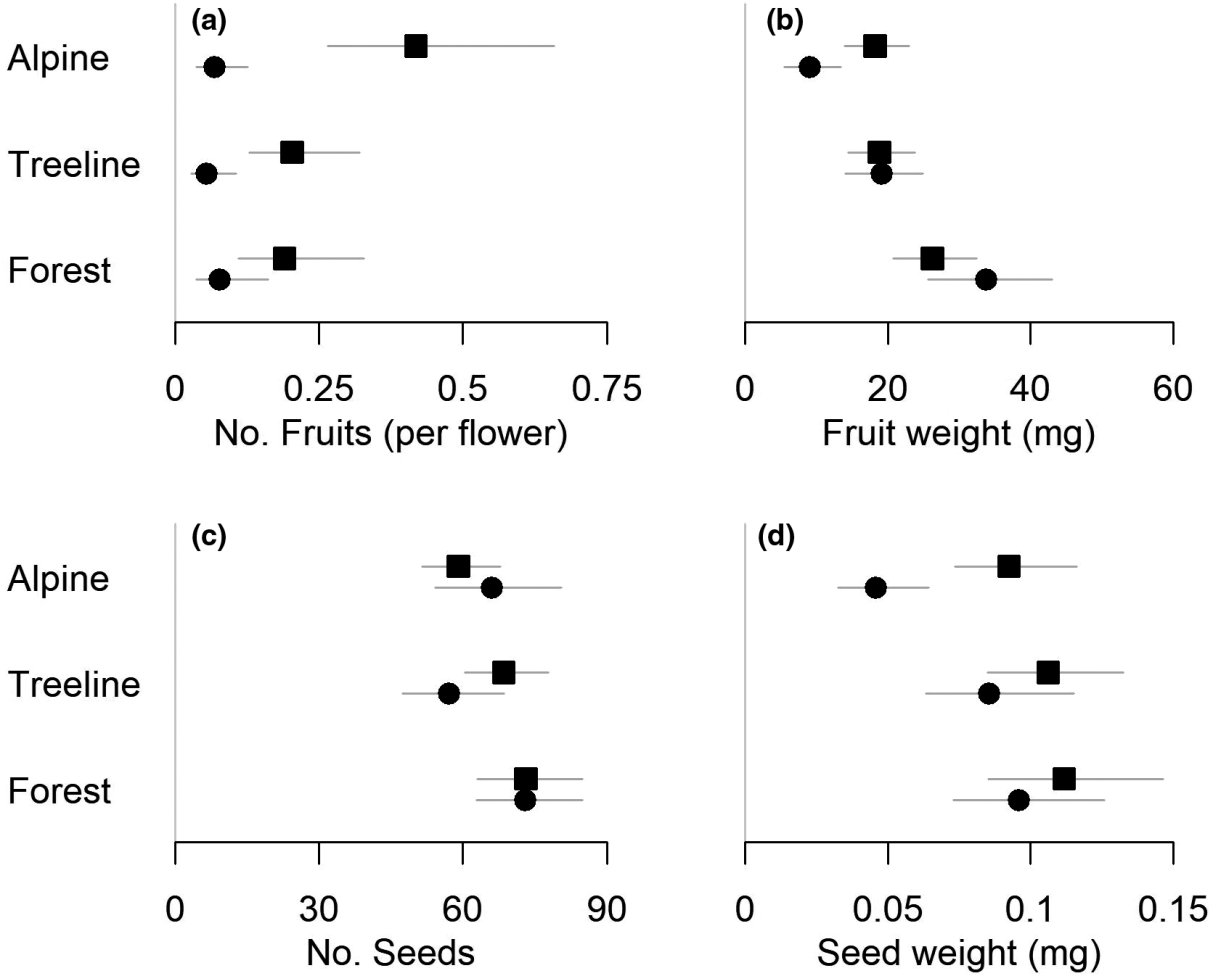
Model estimates ±95% CI for the models in Table 2 for number of fruits (a), fruit weight (mg) (b), number of seeds (c), and seed weight (mg) (d) of *Vaccinium myrtillus* in control (squares) and exclusion (circles) plots in forest, treeline and alpine sites in central Norway. Data on fruit number and fruit weight were collected in 2017 and 2018, whereas data on seed number and seed weight were collected in 2017. The figures show back‐transformed data

Excluding pollinators generally resulted in a significantly lower number of fruits per plot (number of flowers used as an offset; Table 2, Figure 2a). In the alpine sites, the number of fruits was 84% lower in the exclusion plots compared to the control plots. However, this effect was less pronounced in the treeline and forest sites (73% and 59% lower, respectively), as indicated by the interaction effects, of which the forest × exclusion interaction was significant. Under ambient conditions (control plots) the number of fruits was significantly lower in the treeline and forest sites compared to the alpine, suggesting that fewer flowers develop into fruits in these sites even when pollinators are present.

**TABLE 2:** Parameter estimates, standard errors, *z*‐values, and *p*‐values for mixed‐effects models testing the effect of elevation and experimental treatment (control and exclusion) on the number of fruits, fruit weight (mg), number of seeds, and seed weight (mg) of *Vaccinium myrtillus* in forest, treeline and alpine sites in central Norway

	Estimate	Std. Error	*z*‐value	*p*‐value
Number of fruits				
Intercept	−0.87	0.23	−3.77	<.001^***^
Exclusion	−1.81	0.29	−6.28	<.001^***^
Treeline	−0.72	0.20	−3.61	<.001^***^
Forest	−0.79	0.23	−3.41	<.001^***^
Exclusion:treeline	0.49	0.42	1.16	.256
Exclusion:forest	0.92	0.45	2.06	.040^*^
Fruit weight				
Intercept	4.27	0.27	15.81	<.001^***^
Exclusion	−1.26	0.35	−3.55	<.001^***^
Treeline	0.07	0.31	0.23	.815
Forest	0.86	0.33	2.63	.008^**^
Exclusion:treeline	1.29	0.50	2.56	.010^*^
Exclusion:forest	1.94	0.54	3.58	<.001^***^
Number of seeds				
Intercept	4.08	0.07	57.74	<.001^***^
Exclusion	0.11	0.12	0.96	.335
Treeline	0.15	0.09	1.72	.085
Forest	0.21	0.10	2.24	.025^*^
Exclusion:treeline	−0.30	0.16	−1.87	.062
Exclusion:forest	−0.11	0.15	−0.74	.460
Seed weight				
Intercept	−2.38	0.12	−20.44	<.001^***^
Exclusion	−0.70	0.20	−3.56	<.001^***^
Treeline	0.14	0.15	0.94	.346
Forest	0.19	0.17	1.14	.255
Exclusion:treeline	0.48	0.27	1.83	.068
Exclusion:forest	0.55	0.27	2.05	.041^*^

Note

*p*‐values are indicated by asterisks (*p < .05, **p < .01, ***p < .001).

Excluding pollinators resulted in a significantly lower mean fruit weight in the alpine sites, where fruit weight in the exclusion plots was 50% lower than in the control plots (Table 2, Figure 2b). This was, however, not the case in the treeline and forest sites, as indicated by the positive site × exclusion interactions. Further, under both ambient and experimental conditions, fruits were significantly heavier in the forest sites compared to the alpine sites, showing that although fewer fruits are produced in the forest sites, these fruits are larger, regardless of the presence of pollinators.

The number of seeds per fruit under ambient conditions was significantly higher in the forest sites compared to the alpine sites in 2017, with a similar trend for the treeline sites (Table 2, Figure 2c). The exclusion treatment did not have an effect on the number of seeds per fruit, although there was a tendency for a negative effect in the treeline sites, with a 17% lower seed number in the exclusion plots. However, there was a significantly negative effect of pollinator exclusion on the mean weight per seed, showing that pollinator presence affected seed mass rather than seed number (Table 2, Figure 2d). This effect was most pronounced in the alpine, where seed weight in the exclusion plots was 50% lower than in the control plots, and less pronounced in the forest sites, as shown by a significant forest × exclusion interaction, with a similar tendency for the treeline sites.

## 4 DISCUSSION

Excluding pollinators from *Vaccinium myrtillus* resulted in lower fruit production as well as reduced fruit and seed mass, showing that although *V*. *myrtillus* is capable of self‐pollination, cross‐pollination by insects is an important mechanism for seed production. These effects were most pronounced in the alpine tundra, where pollinator exclusion resulted in 84% lower fruit number, 50% lower fruit mass, and 50% lower seed mass. Despite the relatively small sample size of this experiment, the results suggest that the importance of pollinating insects for seed production in this partly selfing plant species is high, especially at high elevation.

Our findings are in line with previous studies showing that exclusion of pollinators from *V*. *myrtillus* reduces seed production (Fröborg, 1995; Nuortila et al., 2002), although the effect can vary between years (Jacquemart, 1997; Jacquemart & Thompson, 1996), confirming that cross‐pollination is indeed important for reproduction in this species. However, the increased importance of pollinators at higher elevations was unexpected given that the contribution of pollinators to plant seed production has been predicted to be higher (e.g., Arroyo et al., 1985; Lázaro et al., 2015; Totland, 1993) or similar (Bingham & Orthner, 1998, see also García‐Camacho & Totland, 2009) in warmer compared to colder climates. Nonetheless, our findings are in line with the genetic study of Wirth et al. (2010) showing that selfing rates in an alpine plant decrease with elevation.

The increasing importance of pollinators with elevation could be due to differences in pollinator activity or efficiency, as well as differences in conspecific pollen transfer (see Ashman et al., 2020), along the elevational gradient. Eckerter et al. (2019) suggest that pollinator visits to *V*. *myrtillus* may correlate positively with light availability, which could explain the higher importance of pollinators in our alpine sites compared to the more shaded treeline and forest sites (see Figure S3). However, Andresen (2019), who studied bumblebees in the *V*. *myrtillus*‐dominated communities in the Grødalen study area, found a higher richness and abundance of bumblebees in the treeline site compared to the forest and alpine. Nonetheless, Andresen (2019) also found a gradient in bumblebee species composition from the forest to the alpine. Moquet et al. (2017) show that the contribution of bumblebees in the pollination of *V*. *myrtillus* differs between species. Differences in bumblebee species composition could therefore explain the higher importance of pollinators for seed production of *V*. *myrtillus* at higher elevations, if higher altitude specialist bumblebee species are more efficient pollinators of *V*. *myrtillus* than lowland generalists.

The higher importance of pollinators at high elevation could also be due to a higher degree of plant adaptation to pollination, either genetically or through phenotypic plasticity. Our study was not designed to assess such differences in plant adaptation between elevational levels, but these factors cannot be ruled out. For instance, Fernández‐Calvo and Obeso (2004) found a change in resource allocation from growth to reproduction in *V*. *myrtillus* with increasing elevation, and Pato and Obeso (2012a) found that fruit mass and seed number of *V*. *myrtillus* increased with altitude up to 100–200 m above the treeline. Similarly, Anadon‐Rosell et al. (2014) show that experimental warming may increase the vegetative growth of *V*. *myrtillus*. Together, these studies suggest a shift in plant resource allocation from reproduction to growth with increasing temperature. Reduced resource allocation to reproduction could explain our findings of the reduced importance of pollination at lower elevations.

Plant clonality could also potentially influence the outcome of our experiment. *V*. *myrtillus* is a highly clonal species, with individual genets being several meters in diameter (Albert et al., 2003, 2004). The clonal structure of this plant suggests that if the fruit set is reduced, for instance, due to experimental treatments, resources within a clone may be allocated to the few flowers which have produced maturing seeds, thereby potentially increasing seed weight and seed number per fruit. Our 1 × 1 m plots were not likely to include entire *V*. *myrtillus* clones, but this did not seem to obscure the effect of the treatment, as both the number of fruits and seed weight were reduced in the pollinator exclusion treatment, indicating no “extra” resource allocation to fruit and seed development.

Although Pato and Obeso (2012b) found no altitudinal difference in the density of flowers or fruits of *V*. *myrtillus*, Boulanger‐Lapointe et al. (2017) found that the number of fruits per flower was twice as high in the forest compared to alpine habitats. In contrast, our results show a significantly lower number of fruits per flower in the control plots in the forest and treeline sites compared to the alpine sites. The generally lower fruit set at low elevation may be due to elevational differences in pollinator activity and efficiency, or plant adaptations, as discussed above. However, in our opinion, it is more likely due to an *Epirrita* outbreak, which had a strong defoliating effect on *V*. *myrtillus* in the forest and some of the treeline sites in 2017. Boulanger‐Lapointe et al. (2017) show that the number of *V*. *myrtillus* flowers, which in turn determines the number of fruits, is correlated with *Epirrita* outbreaks in northern Finland. We observed *Epirrita* larvae grazing on leaves and flowers of *V*. *myrtillus* both inside and outside the exclusion cages. Thus, it is unlikely that the *Epirrita* outbreak contributed to a less pronounced difference between the control and exclusion treatment in the forest sites. Nonetheless, grazing by *Epirrita* larvae may have contributed to the strong gradient in fruit production from the forest to the alpine sites under ambient conditions.

## 5 CONCLUSION

Our findings show that the importance of insect pollination for seed production in *V*. *myrtillus* varies along elevational gradients, and thus with mean summer temperatures, meaning that these plant–pollinator interactions may be affected by climate change. However, the reduced importance of pollinators at low elevation suggests that *V*. *myrtillus* may be more capable of compensating for pollinator loss by self‐pollination in warmer compared to colder climates. Moreover, the fact that this generalist plant species are visited by many different pollinator species (Andresen, 2019), suggests that it is probably robust to climate‐driven changes in the pollinator community (e.g. Hegland et al., 2009; Rafferty, 2017). *V*. *myrtillus* is an important resource for many pollinator species depending on early‐flowering plants (Moquet et al., 2015), including in our study area (Andresen, 2019). Thus, the robustness of *V*. *myrtillus* to changes in plant–pollinator interactions indicates that a climate‐driven mismatch between *V*. *myrtillus* and its pollinators may have greater consequences for the insects than the plant. However, a change in the outcrossing rate of *V*. *myrtillus* due to climate warming, could affect populations’ genetics, which in turn may influence population viability and adaptability.

The climate‐driven upward shifts in elevation of plants (e.g., Grabherr et al., 1994; Pauli et al., 2012; Steinbauer et al., 2018), which is also found in the Grødalen study area (Løkken et al., 2020), are likely driven mainly by seed‐dispersal rather than clonal propagation, as clonal growth is a primarily local phenomenon. This implies that unless pollinator communities shift in synchrony with plant communities, lack of pollination may slow down the upward shift of plants. In the case of *V*. *myrtillus*, our findings suggest that changes in the pollinator community will not greatly reduce seed production in a warmer climate. Thus, the climate‐driven upslope movement of the species should not be impeded by a lack of seeds.

Climate change will, however, not only result in increased mean temperatures but also more frequent climate extremes. Extreme warming events may influence the pollinator community (Zoller et al., 2020) as well as plants (Orsenigo et al., 2014). The summer of 2018 was unusually warm in our study area, coinciding with the production of fewer fruits per flower of *V*. *myrtillus* than in 2017. However, further research is needed to disentangle the direct effects of extreme warming on plant reproduction from indirect effects due to changes in the pollinator community, as well as explore the generality of these mechanisms.

## AUTHOR CONTRIBUTIONS

**Siri L. Olsen:** Conceptualization (equal), Formal analysis (lead), Investigation (equal), Methodology (equal), Writing—original draft (lead), Writing—review & editing (lead). **Marianne Evju:** Conceptualization (equal), Investigation (equal), Methodology (equal), Project administration (lead), Writing—original draft (supporting), Writing—eview & editing (equal). **Jens Åström:** Conceptualization (equal), Formal analysis (supporting), Investigation (equal), Methodology (equal), Writing—original draft (supporting), Writing—review & editing (equal). **Jørn Olav Løkken:** Investigation (equal), Writing—original draft (supporting), Writing—review & editing (equal). **Sondre Dahle:** Investigation (equal), Writing—original draft (supporting), Writing—review & editing (equal). **Jonas L. Andresen:** Investigation (equal), Writing—original draft (supporting), Writing—review & editing (equal). **Nina E. Eide:** Conceptualization (equal), Investigation (equal), Writing—original draft (supporting), Writing—review & editing (equal).

## CONFLICT OF INTEREST

The authors declare no conflict of interest.

## Supporting information

Figure S1

Figure S2

Figure S3

Figure S1‐S3

## Data Availability

The data is available at the Dryad Digital Repository: https://doi.org/10.5061/dryad.t1g1jwt4m
