# Supplementary figures and images for: Climate influence on plant–pollinator interactions in the keystone species Vaccinium myrtillus
Source: Ecol Evol. 2022 May 23;12(5):e8910. doi: 10.1002/ece3.8910 (PMC9126989; doi:10.1002/ece3.8910)

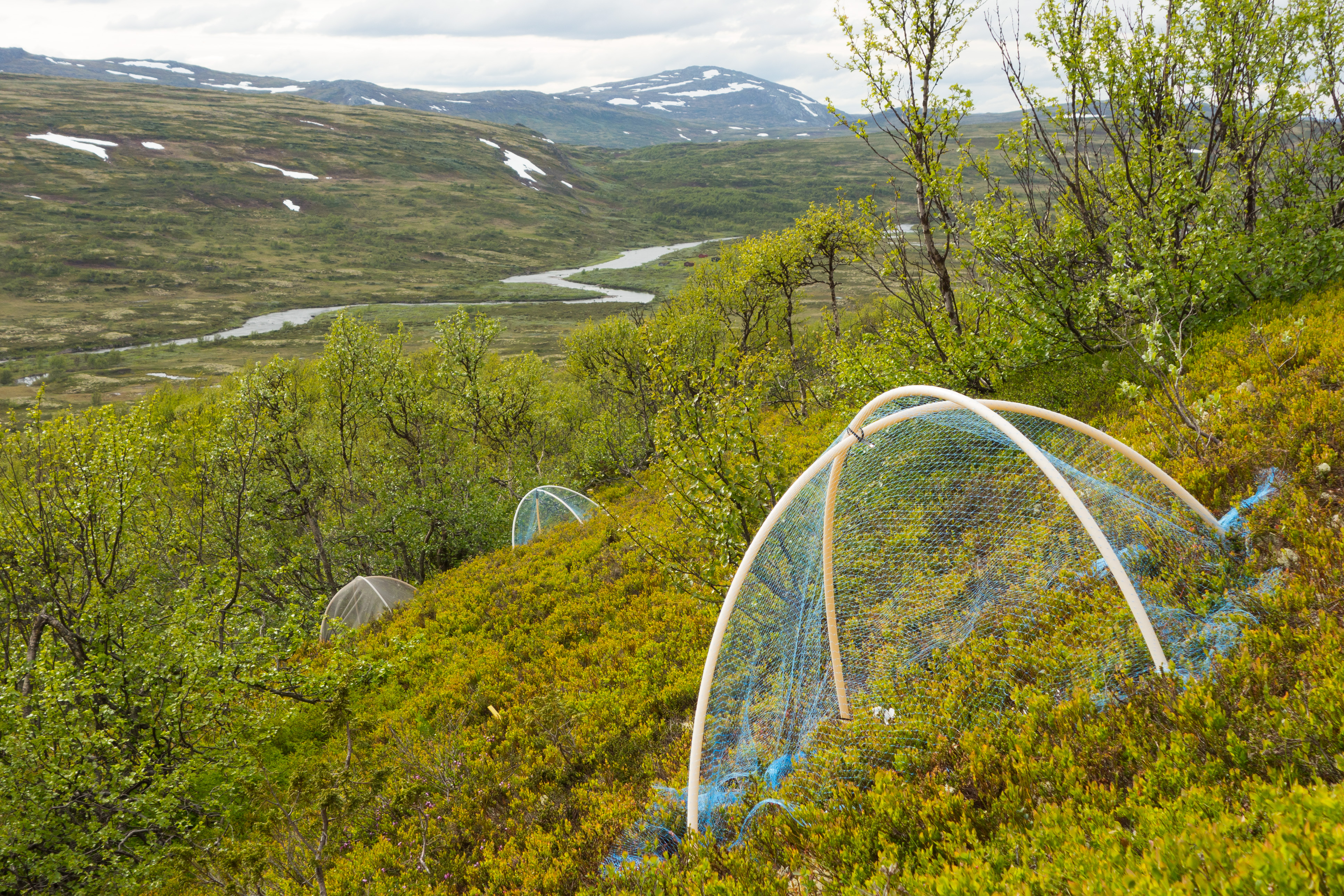

Supplement: Supplementary file 1 — Figure S1 [file ECE3-12-e8910-s002.jpg]

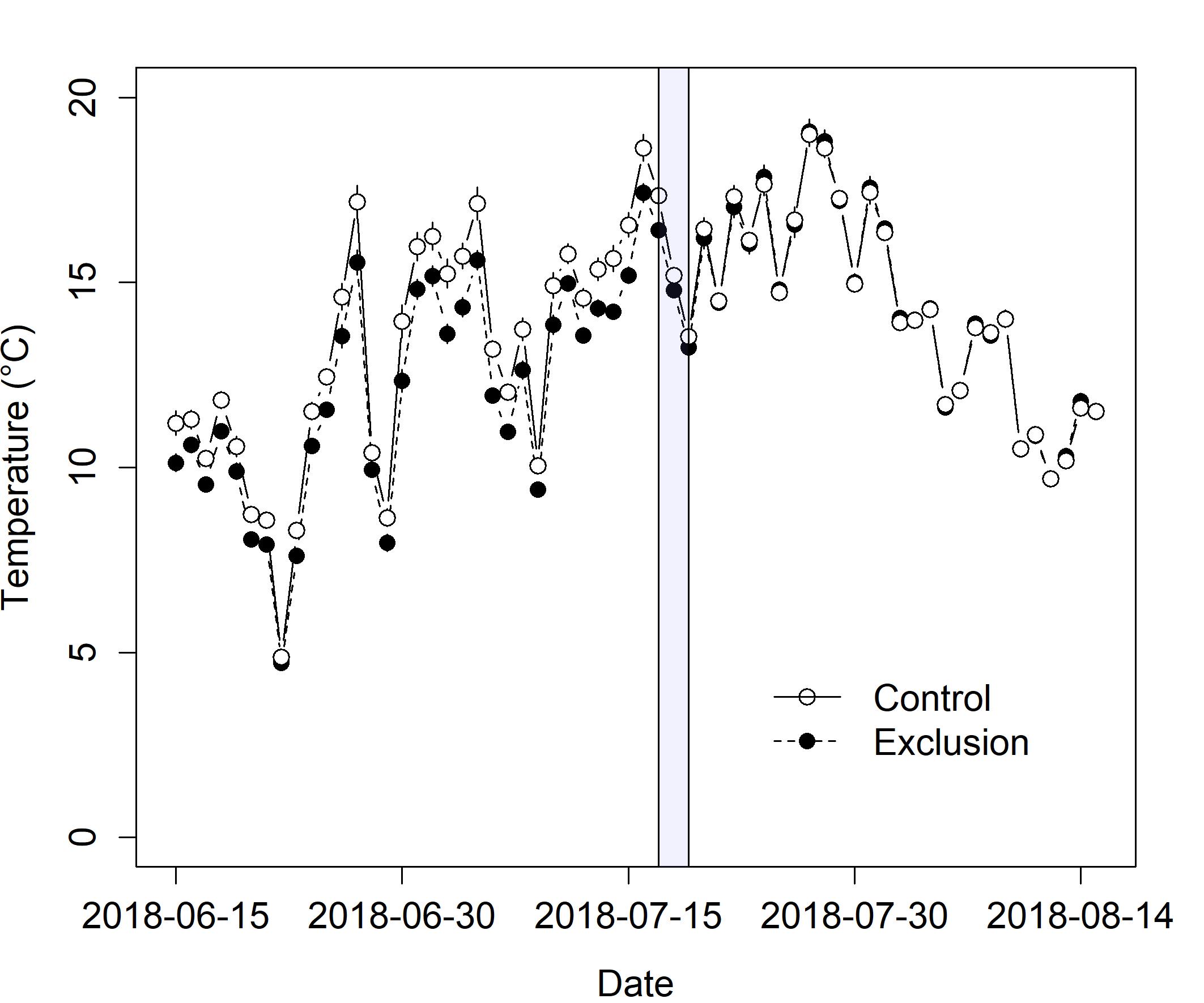

Supplement: Supplementary file 2 — Figure S2 [file ECE3-12-e8910-s004.jpg]

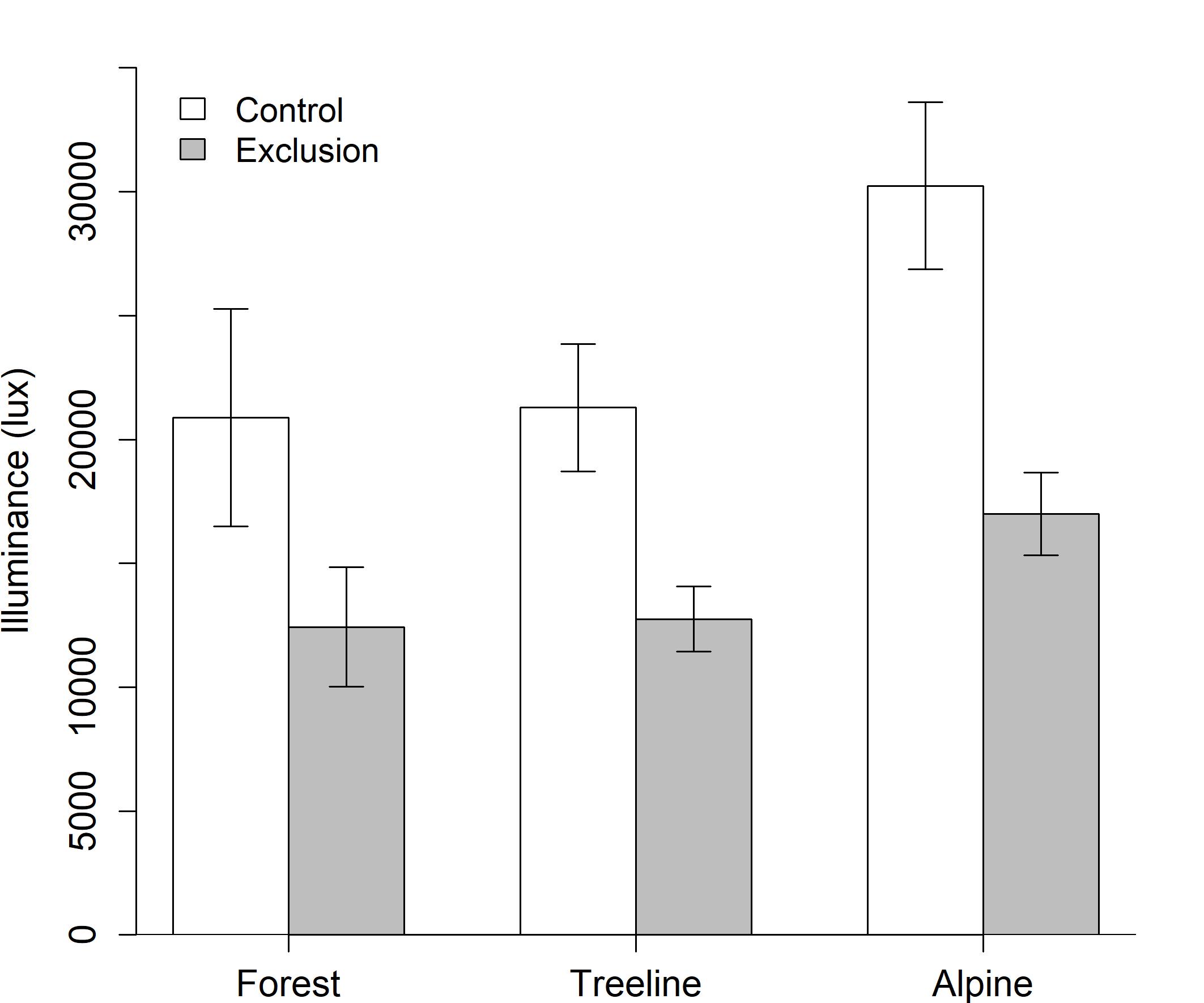

Supplement: Supplementary file 3 — Figure S3 [file ECE3-12-e8910-s001.jpg]
