# Supplementary material for: Climate influence on plant–pollinator interactions in the keystone species Vaccinium myrtillus
Source: Ecol Evol. 2022 May 23;12(5):e8910. doi: 10.1002/ece3.8910 (PMC9126989; doi:10.1002/ece3.8910)
Supplement: Supplementary file 4 — Figure S1‐S3 [file ECE3-12-e8910-s003.docx]

Appendix


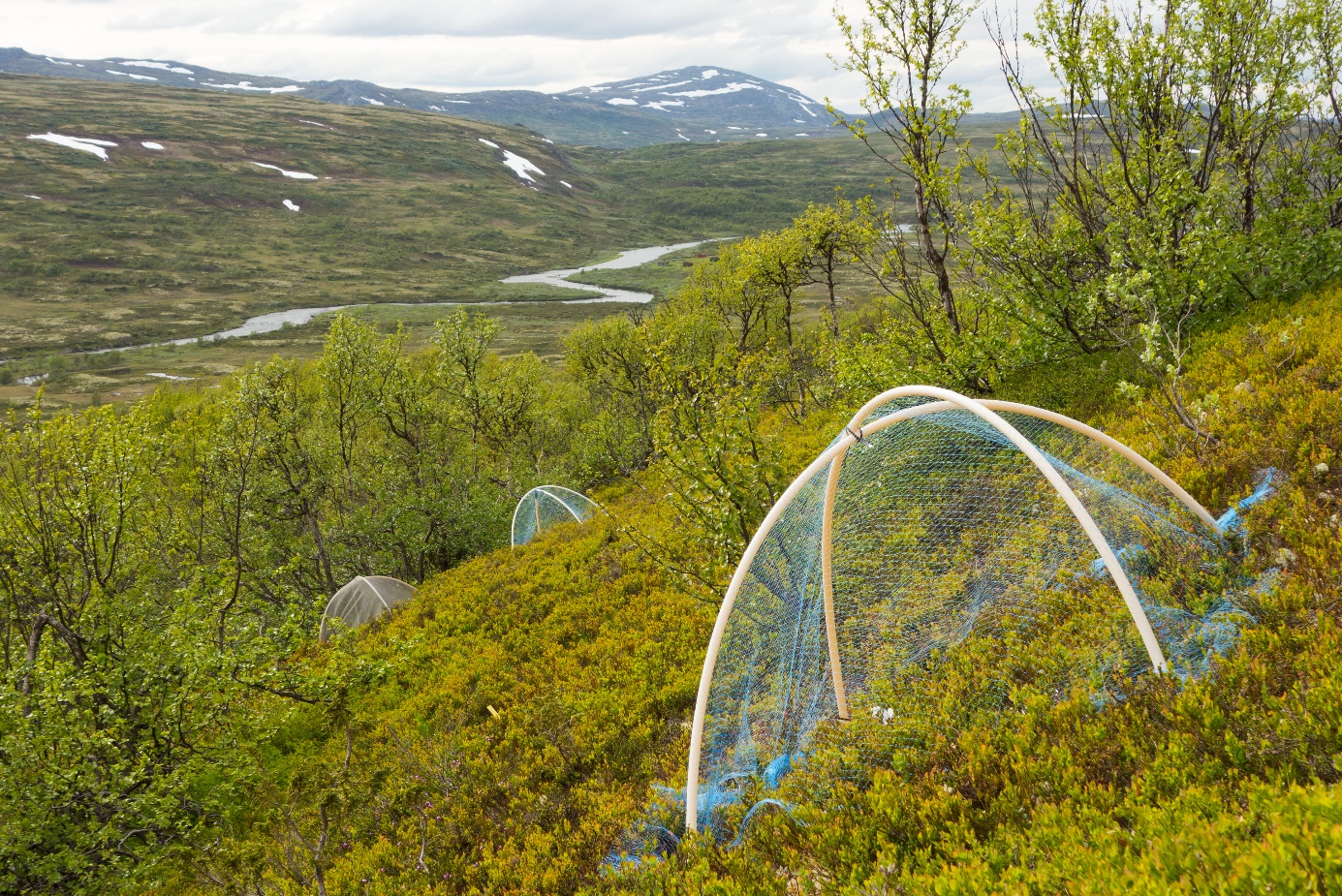


***Fig. A1.*** *The dome-shaped cages used in the pollinator reduction and exclusion treatments. Each cage was made of two approximately 2.5 m long PVC tubes bent diagonally over the plots and covered in berry netting (reduction treatment) or insect netting (exclusion treatment). Photo: S. Dahle.*


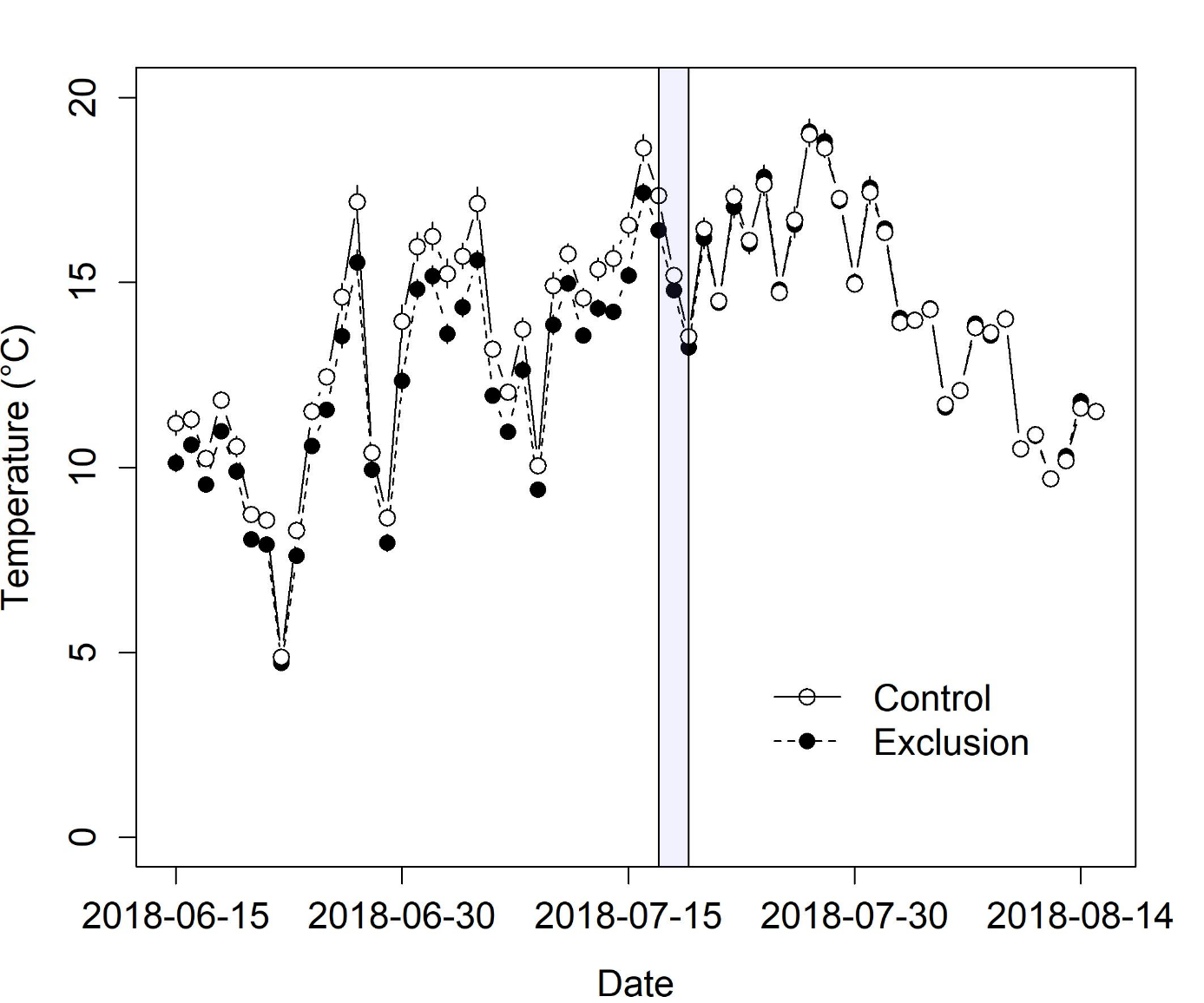


***Fig. A2.*** *Mean temperature (°C) ± 1SE in control (open circles) and exclusion (black circles) plots in forest, treeline and alpine sites in central Norway from 15 June to 15 August 2018 before (left) and after (right) the removal of the pollinator exclusion cages. The period of cage removal is indicated by the grey bar.*


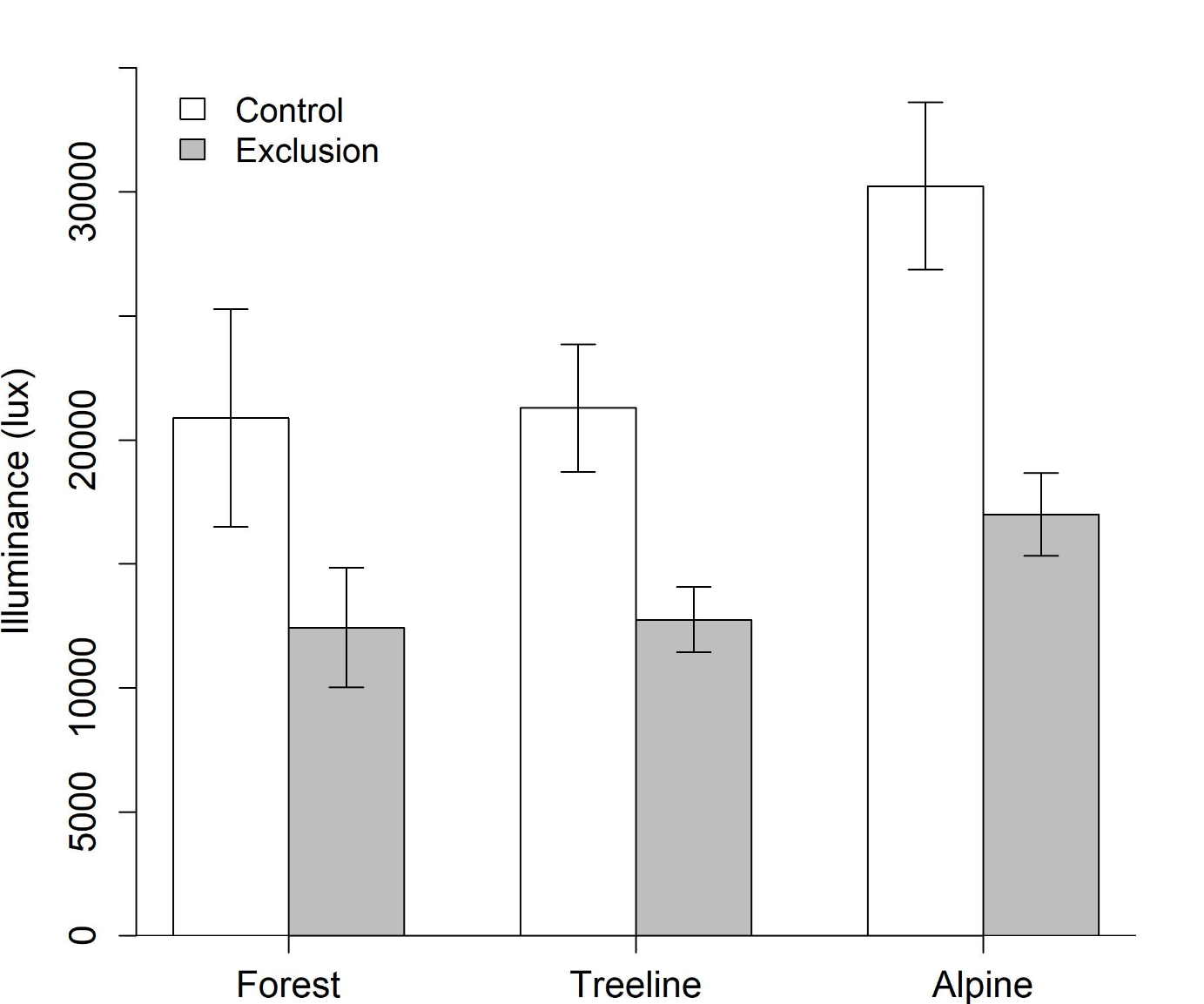


***Fig. A3.*** *Mean illuminance (lux) ± 1SE in control and exclusion plots in forest, treeline and alpine sites in central Norway in 2018.*
